# Graphical approaches for the control of generalized error rates

**DOI:** 10.1002/sim.8595

**Published:** 2020-06-17

**Authors:** David S. Robertson, James M. S. Wason, Frank Bretz

**Affiliations:** 1 MRC Biostatistics Unit, University of Cambridge, Cambridge, UK; 2 Institute of Health and Society, Newcastle University, Newcastle, UK; 3 Statistical Methodology, Novartis Pharma AG, Basel, Switzerland; 4 Section for Medical Statistics, Medical University of Vienna, Vienna, Austria

**Keywords:** false discovery proportion, generalized familywise error rate, hypothesis testing, multiple comparison procedures, multiple endpoints

## Abstract

When simultaneously testing multiple hypotheses, the usual approach in the context of confirmatory clinical trials is to control the familywise error rate (FWER), which bounds the probability of making at least one false rejection. In many trial settings, these hypotheses will additionally have a hierarchical structure that reflects the relative importance and links between different clinical objectives. The graphical approach of Bretz et al (2009) is a flexible and easily communicable way of controlling the FWER while respecting complex trial objectives and multiple structured hypotheses. However, the FWER can be a very stringent criterion that leads to procedures with low power, and may not be appropriate in exploratory trial settings. This motivates controlling generalized error rates, particularly when the number of hypotheses tested is no longer small. We consider the generalized familywise error rate (*k*-FWER), which is the probability of making *k* or more false rejections, as well as the tail probability of the false discovery proportion (FDP), which is the probability that the proportion of false rejections is greater than some threshold. We also consider asymptotic control of the false discovery rate, which is the expectation of the FDP. In this article, we show how to control these generalized error rates when using the graphical approach and its extensions. We demonstrate the utility of the resulting graphical procedures on three clinical trial case studies.

## 1 Introduction

In modern clinical trials, it is increasingly common to test multiple hypotheses simultaneously. This multiplicity is driven by evaluating multiple therapies in parallel, the identification of multiple subgroups and the measurement of multiple endpoints. Given that these multiple hypotheses are assessed simultaneously, there is a strong emphasis on controlling the total number or proportion of false positives (ie, type I errors) in some way. Indeed, for confirmatory clinical trials, regulatory guidelines state that the familywise error rate (FWER) should be strongly controlled.^1,2^ This ensures that the maximum probability of making at least one type I error is below some prespecified level (under any configuration of the parameters being tested).

The increase in multiplicity in clinical trials also tends to go hand-in-hand with an increase in the complexity of the objectives and structure of the hypotheses tested. A key setting where this occurs is when measuring multiple endpoints to answer distinct (but related) clinical questions. The corresponding hypotheses often fit naturally within a hierarchical structure that reflects the relevant importance and links between the clinical questions that the trial aims to answer. For example, in a trial with both a primary and secondary hypothesis, the trialist may only wish to test the secondary hypothesis if the primary hypothesis is first rejected. More complex hierarchical structures can be formed as the number of hypotheses increases.

Many methods have been developed for FWER control that respect complex trial objectives and multiple structured hypotheses. A highly flexible framework for doing so is the graphical approach to hypothesis testing, as proposed independently by Bretz et al^3^ and Burman et al.^4^ In the framework of Bretz et al,^3^ vertices represent the null hypotheses and weights represent the local significance levels, which are propagated through weighted, directed edges. The resulting multiple testing procedure can be tailored to structured families of hypotheses with arbitrary dependence between the hypotheses, and allows the visualization of complex decision strategies in an easily communicable way. Many well-known procedures for FWER control are special cases of the graphical approach, such as the fixed sequence (or hierarchical) test,^5^ the Holm procedure,^6^ the Hochberg procedure,^7^ and several gatekeeping procedures.^8-10^

However, controlling the FWER is a very stringent criterion, especially as the number of hypotheses increases. By controlling the probability of even a single type I error, the power of FWER-controlling procedures can be very low, with little chance of any of the individual hypotheses being rejected. While strong FWER control is appropriate in confirmatory contexts, in exploratory trial settings such strict criterion may not be necessary. Indeed, as reflected in the FDA (2017) guidance on multiple endpoints in clinical trials,^1^ exploratory analyses can be included in a trial to explore and generate new hypotheses. Since such exploratory hypotheses will often be followed up with confirmatory testing, strict FWER control at the exploratory stage is no longer necessary.

Westfall and Bretz^11^ expand on this argument, by categorizing the hypothesis tests in a typical clinical trial into families of “efficacy,” “safety,” and “exploratory” tests. For the efficacy family, the primary endpoints and main secondary endpoints are the basis of regulatory approval and labeling, and hence require strong FWER control. However, there may also be “lesser interest tests” (eg, multiple time point analyses), where FWER controlling methods are not needed. Nonetheless, the authors note that some form of multiplicity adjustment would strengthen the claims made for this set of tests. For the safety family, serious and known treatment-related adverse events (AEs) do not require multiplicity adjustment (since type II errors are of much greater concern). However, for all other AEs, the authors state that there is a clear need to recognize the multiplicity problem, and note that the use of the false discovery rate (FDR) may be more appropriate here. Finally, for the family of exploratory tests (which may include both safety and efficacy tests), the authors state that “standard multiplicity adjustment here seems unreasonable, as power will be very low,” and again recommend the use of FDR controlling methods.

All this demonstrates that outside of the context of testing the primary and main secondary endpoints for regulatory approval and labeling, strong FWER control may not be needed, even in confirmatory trials. Less stringent error rates can then be used, where more than one false rejections are acceptable in order to increase the power of the trial. One approach is to control the generalized FWER, or *k*-FWER. The *k*-FWER is the probability of making at least *k* false rejections, where *k* ≥ 1. Clearly the FWER is a special case of the *k*-FWER when *k* = 1. A number of methods controlling the *k*-FWER have been proposed, including step-up procedures^12-15^ and permutation-based procedures.^16-18^ Another approach is to accept a certain proportion of false rejections, that is, to control the false discovery proportion (FDP). The FDP is closely related to the well-known FDR,^19^ which is now a common error rate to control in experiments with a large number of hypotheses, such as genomic studies. The FDR is the expected value of the FDP, that is, the FDR is the expected proportion of errors among the rejected hypotheses. Although controlling the FDR controls the expectation of the FDP, in practical applications the actual FDP might be far from its expectation.^20^ In the context of clinical trials with a relatively small number (< 100) of hypotheses, this motivates control of the tail probability of the FDP and hence guaranteeing control over the probability of having a high proportion of false discoveries. Some methods for controlling the FDP have previously been proposed.^12,17,21^

In general, the various procedures proposed in the literature for generalized error rate control are not suitable for structured hypothesis testing problems encountered in the context of clinical trials, as they do not respect the underlying hierarchical structure of the testing strategy. In order to do so, in this article we show how to control both the *k*-FWER and FDP (as well as asymptotic control of the FDR) when using the graphical approach of Bretz et al^3^ and its extensions. We achieve this by modifying and applying the methodology for *k*-FWER and FDP control given by van der Laan et al^22^ and Romano and Wolf^18^ to the graphical framework. The performance of the resulting procedures are compared analytically and through simulations in the context of various case studies.

The rest of the article is structured as follows. In Section 2, we introduce the basic notation and the graphical approach to hypothesis testing. Section 3 shows how to modify the graphical approach to control the *k*-FWER, while Section 4 gives a further modification of the graphical approach to control the FDP as well as (asymptotic) control of the FDR. Section 5 shows how to use the proposed procedures for a number of extensions to the graphical approach. We illustrate the proposed methods using three case studies in Section 6, and conclude with a discussion in Section 7.

## 2 Graphical Approach To Hypothesis Testing

Consider simultaneously testing multiple null hypotheses *H*_1_,…,*H_m_*which are related in some way and so can be thought of as a family of hypothesis tests. Since we are jointly testing multiple hypotheses, there is a resulting multiplicity problem that we wish to take account of in the testing procedure. The standard approach for confirmatory clinical trials is to control the FWER (in the strong sense) below some prespecified level *α*, where *α* ∈ (0,1). That is, *P*(*V* > 0) ≤ *α* under any configuration of true and false null hypotheses, where *V* denotes the number of false rejections made. We consider testing *H*_1_,…,*H_m_* using the corresponding *P*-values *P*_1_,…,*P_m_*. Let *M* = {1,… ,*m*} denote the associated index set and assume that the *P*-values associated with the true null hypotheses satisfy *P*(*p_i_* ≤ *u*) ≤*u* for any *u* ∈ [0,1].

We now describe the graphical approach to hypothesis testing introduced by Bretz et al,^3^ which controls the FWER. In this approach, the hypotheses *H*_1_,…,*H_m_* are represented by vertices, with associated weights denoting the significance levels. Any two vertices *H_i_*and *H_j_* are connected by a directed edge with weight *g_ij_*, which indicates the fraction of the significance level *α_i_* which is propagated from *H_i_* to *H_j_* if *H_i_* is rejected. If *g_ij_* = 0 then there is no propagation of the significance levels, and the edge can be dropped for convenience from the graphical visualization. These *g_ij_* form an *m* × *m* transition matrix ***G*** = (*g_ij_*), which fully characterizes the propagation of significance levels.

**Figure 1:**
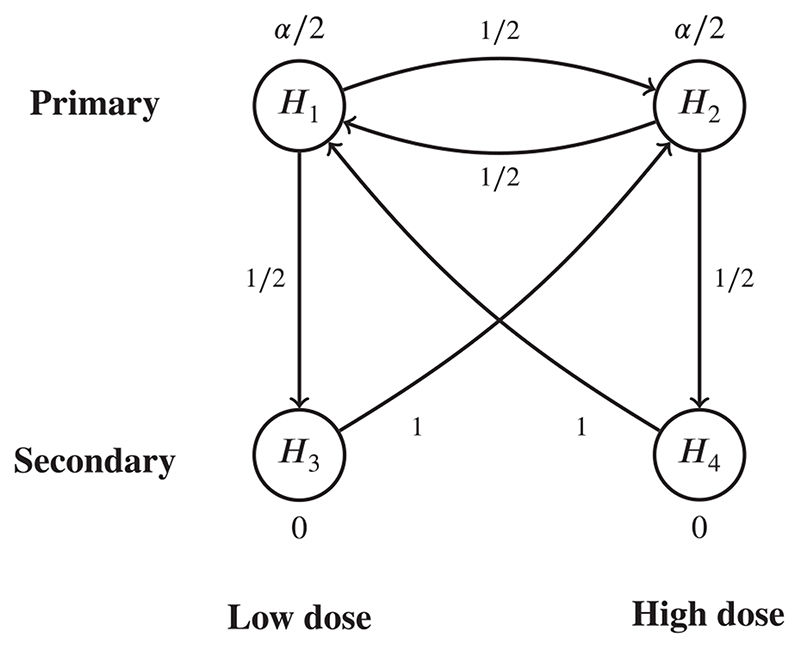
Graph showing a possible testing strategy for a diabetes trial with a primary and secondary endpoint that tests two doses of a drug against a placebo, as given in Maurer et al^23^

As an example, consider a trial in diabetes patients that compares two doses (a low dose and a high dose) of an experimental drug against placebo, in terms of both a primary and secondary clinical endpoint. Since the primary endpoint is more important than the secondary one, the trialist tests the primary hypothesis first; only if this is rejected is the secondary hypothesis then tested. Assuming that both doses are equally important, a possible testing strategy is shown in the graph in Figure 1, as given in Maurer et al.^23^

Bretz et al^24^ proposed a graphical weighting strategy which allows the computation of the set of weights for any intersection hypotheses *H_J_* = ⋂_*j∈J*_ *H_j_*, *J* ⊆ *M*. The graphical weighting strategy requires the specification of initial weights *w_i_*(*M*), *i* ∈ *M*, for the global null hypothesis *H_M_* and the transition matrix ***G***, with entries *g_ij_* satisfying the regularity conditions

(1) $$0\leq g_{ij}\leq 1,\mathrm{and}g_{ii}=0\mathrm{and}\text{and}\;\overset{m}{\underset{j=1}{\sum^{\text{​}}}}g_{ij}\leq 1\mathrm{and}\text{for }\;\text{all}\;i,j\in M.$$

Algorithm 7 in Appendix A1 reproduces the algorithm given in Bretz et al^24^ for calculating the weights *w_j_*(*J*), *j* ∈ *J*, which can then be used for testing the intersection hypothesis *H_J_*.

Given these weights, a weighted multiple testing procedure can then be applied to each intersection hypothesis *H_J_*, such as a weighted Bonferroni test, or a weighted parametric test if the joint distribution of the *P*-values is known.^24^ Applying a weighted Bonferroni test is the simplest option, and leads to the original Bonferroni-based graphical approach for FWER control based on a shortcut procedure where the *m* hypotheses can be tested sequentially, and hence requires at most *m* steps of the algorithm^3^ (see also Algorithm 8 in Appendix A1).

Adjusted *P*-values can also be calculated when using this graphical approach, which then allow the hypothesis tests to be easily performed at any significance level *α*. More formally, the adjusted *P*-value $P_{j}^{\text{adj}}$ for hypothesis *H_j_* is the smallest significance level at which one can reject the hypothesis using the given multiple test procedure.^3^ Algorithm 9 in Appendix A1 reproduces the algorithm given in Bretz et al^3^ for calculating adjusted *P*-values.

The R package gMCP^25^ provides functions and a graphical user interface to perform all of the calculations described above.

## 3 Graphical Approaches for *K*-Fwer Control

Controlling the *k*-FWER at prespecified level *α* implies that *P*(*V* > *k*) ≤ *α*, where *V* is the number of false rejections. The generalized Bonferroni procedure controls the *k*-FWER:^12,26^ Reject any *H_i_* for which *p_i_* ≤ *kα*/*m*. Assuming known positive weights *w_i_* that satisfy $\sum_{i=1}^{m}w_{i}=1$, Romano and Wolf^18^ introduced the weighted generalized Bonferroni procedure: Reject any *H_i_* for which *p_i_* ≤ *w_i_kα*. If *w_i_* = 1/*m* for all *i* then this is equivalent to the unweighted version.

In order to extend the graphical approach for controlling the *k*-FWER, it is tempting to simply replace *α* by *kα*, in analogy to the modification made for the generalized Bonferroni procedures. However, in general this does not control the *k*-FWER for *k* > 1. As a counterexample, consider the Holm procedure with *m* hypotheses, which can be represented as a graph with initial weights *w_i_*(*M*) = 1/*m* and *g_ij_* = 1/(*m* − 1) for all *i*,*j* ∈ *M*, *i* ≠ *j*. Using the graphical weighting strategy (Algorithm 7), we have *w_i_*(*I*) = 1/|*I*| for all *i* ∈ *I* and *I* ⊆ *M*. Replacing *α* by *kα* in the graphical approach (Algorithm 8) is hence equivalent to a stepdown procedure where the *i*th smallest *P*-value is compared with the significance level $\alpha_{i}=\frac{k\alpha }{m+1-i}$ . However, since $\frac{k\alpha }{m+1-i}>\frac{k\alpha }{m+k-i}$ for *k* > 1, the result of Theorem 2.3 in Lehmann and Romano^12^ shows that this procedure does not control the *k*-FWER. Hence, we turn to alternative procedures for *k*-FWER control.

### 3.1 Augmented graphical approach for *k*-FWER control

We first consider a simple method of controlling the *k*-FWER described in van der Laan et al [22, Procedure 1], which can be applied to give a graphical approach for *k*-FWER control. The original method starts with an initial procedure that controls the usual FWER, and then augments this by additionally rejecting the hypotheses associated with the smallest *k* − 1 remaining (unrejected) *P*-values. These *k* − 1 additionally rejected hypotheses can be freely chosen, and so we aim to respect the hierarchical structure of the underlying multiple testing problem and to avoid rejecting hypotheses with large *P*-values. This results in the following augmented graphical approach for *k*-FWER control.

**Algorithm 1 (Augmented graphical approach for *k*-FWER control).**

(i) *Apply the usual Bonferroni-based graphical procedure for FWER control given in Algorithm 8*.
(ii) *Let I denote the index set of any remaining (unrejected) hypotheses. If I is empty then stop; otherwise continue with steps (ii) to (iv) of Algorithm 8 with α replaced by α, until up to k* − 1 *additional (augmented) rejections are made*.

Here *δ* ≥ 0 determines how many of the “free” rejections we use, and can be set larger than *α*. In fact, we can even set *α* large enough to ensure that *k* − 1 additional hypotheses are rejected, regardless of the observed *P*-values (see below). Of course, this comes at the potential cost of rejecting hypotheses with *P*-values close to 1 that are likely to be null. Conversely, low values of *δ* mean that we are only willing to reject a hypothesis if it has reasonably substantial evidence against it.

In step (ii) of Algorithm 8, there may be a choice as to which of the hypotheses *j* ∈ *I* to reject. Since there can only be a maximum of *k* additional rejections in step (ii) of Algorithm 1, the order in which hypotheses are rejected does matter here. One sensible choice is to set *j* = arg min_*i*∈*I*_{*p_i_*/*w_i_*(*I*)}, which we use in the remainder of the article.

The choice of *δ* can be data-dependent to ensure that (up to) *k* − 1 additional rejections are made. More explicitly, we can increase *δ* so that one additional rejection is made, then if necessary increase *δ* until another additional rejection is made, and so on. This allows an alternative formulation of the augmented graphical approach based on adjusted *P*-values, which does not depend on an explicit choice of *δ*.

**Algorithm 2 (Adjusted augmented graphical approach for *k*-FWER control).**

(i) *Calculate the m adjusted P-values $P_{i}^{\text{adj}\;}$ corresponding to the usual Bonferroni-based graphical procedure for FWER control, as detailed in Algorithm 9*.
(ii) *Reject all hypotheses H_i_ with* $P_{i}^{\text{adj}\;}\leq \alpha$.
(iii) *Let I denote the index set of any remaining (unrejected) hypotheses. If I is empty then stop; otherwise order the remaining hypotheses in nondecreasing order*: $P_{\left(1\right)}^{\text{ad}]}\leq \ldots \leq p_{\left(|I|\right)}^{\text{adj}}$
(iv) *Additionally reject up to A* = *min* (|I|, *k* − 1) *hypotheses H*_(*i*)_ *corresponding to* $p_{\left(1\right)}^{\text{adj}\;},\ldots ,P_{\left(A\right)}^{\text{adj}\;}$

If there are ties in the ordering in step (iii), they can be broken by choosing the hypothesis with the smallest index, for example. Algorithm 1 will give the same rejections as Algorithm 2 for *δ* large enough. In addition, the R package gMCP^25^ can straightforwardly be used to implement Algorithm 1 in two stages corresponding to steps (i) and (ii). Hence, we focus on Algorithm 1 in the rest of the article.

**Figure 2:**
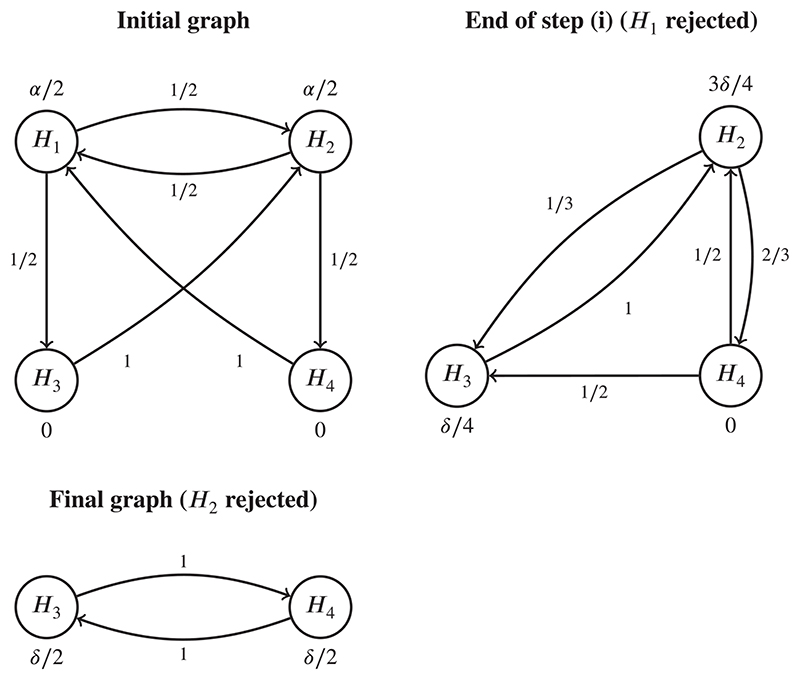
The augmented graphical approach for *k*-FWER control applied to the diabetes trial. FWER, familywise error rate

**Example 1** (Example of the augmented graphical approach for *k*-FWER control:). Consider the graph of the diabetes trial given in Figure 2, where we control the *k*-FWER for *k* = 2 with *α* = .05. Suppose also that the *P*-values are given by *P*_1_ = .01, *P*_2_ = .03, *P*_3_ = .02, *P*_4_ = .024.

In step (i) of Algorithm 1, the usual Bonferroni-based graphical procedure for FWER control would only reject *H*_1_. The updated graph (ie, removing node *H*_1_ and propagating the local significance levels) is then used in step (ii) of Algorithm 1, with *α* replaced by *δ*. Supposing that *δ* = 0.5, we would then reject *H*_2_. At this point, we have made *k* − 1 additional rejections, and so we stop testing having rejected *H*_1_ and *H*_2_. Figure 2 demonstrates each step of the augmented procedure graphically.

### 3.2 Generalized graphical approach for *k*-FWER control

As an alternative approach, we focus on Algorithm 7, which gives weights *w_j_*(*J*), *j* ∈ *J*, for any *J* ⊆ *M*. As shown in Bretz et al,^3^ these weights satisfy the monotonicity condition

(2) $$w_{j}(J)\leq w_{j}\left(J^{\prime }\right)\mathrm{and}\text{for}\;\text{all}\;J^{\prime }\subseteq J\subseteq M\text{and}\;j\in J^{\prime }.$$

Hence, we can apply the generic stepdown method for *k*-FWER control described in Romano and Wolf [18, Algorithm 4.1] with these weights to give the following generalized Bonferroni-based algorithm. Essentially, we simply set the critical constants ${\hat{c}}_{n,K,i}(1-\alpha ,k)$ in their algorithm equal to *w_i_*(*K*), where *K* is used in step (iv) of Algorithm 3 below to index the subsets including *k* − 1 of the previously rejected hypotheses. In what follows, we refer to this as the generalized graphical approach for *k*-FWER control.

**Algorithm 3 (Generalized graphical approach for *k*-FWER control).**

(i) *Set I* = *M*.
(ii) *Reject any H_i_*, *i* ∈ *I for which p_i_* ≤ *w_i_*(*I*)*kα*
(iii) *Let R* = {*i* ∈ *I*:*p_i_* ≤ *w_i_*(*I*)*kα*}*. If* |*R*| *< k or* |*R*| = |*I*| *then stop; otherwise update I* → *I*\*R*.
(iv) *Reject any H_i_*, *i* ∈ *I for which*
  $$p_{i}\leq \underset{J\subseteq R,|J|=k-1}{\min}\left\{w_{i}(K):K=I\cup^{\text{​}}J\right\}k\alpha$$
  *If no such H_i_ exists then stop; otherwise let R* ^′^ *be the indices of these rejected hypotheses*.
(v) *Update the sets I and R as follows:*
  $$\begin{array}{c} I\to I\setminus R^{\prime }\\ R\to R\cup^{\text{​}}R^{\prime } \end{array}$$
(vi) *If* |*I*| ≥ 1*, go to step (iv); otherwise stop*.

In Algorithm 3, at each step *R* is simply the set of indices of all the hypotheses that have been rejected previously, and *I* is the set of indices of the remaining hypotheses *M*\*R*. The algorithm is in a similar spirit to the graphical weighting strategy,^24^ in the sense that there is a separation between the weighting strategy and the graphical test procedure which allows the generalization to *k*-FWER control.

In step (iii) of Algorithm 3, if |*R*| *< k* − 1 and |*R*| ≠ |*I*| then we can freely reject additional hypotheses so that a total of (up to) *k* − 1 rejections are made, while still controlling the *k*-FWER, since the algorithm will stop at this step. In order to respect the hierarchical structure of the underlying multiple testing procedure, and to avoid rejecting hypotheses with large *P*-values, we propose the following subprocedure in step (iii) if |*R*| *< k* − 1:

1. Set *I* → *I*\*R* and follow steps (ii) to (iv) of the usual Bonferroni-based graphical procedure for FWER control (Algorithm 8) with *α* replaced by *δ*, until up to *k* − 1 additional rejections have been made.

As before, *α* ≥ 0 determines how many of the “free” rejections we use, and hence can be set larger than *α* or made data-dependent so that (up to) *k* − 1 additional rejections are made.

Looking at Algorithm 3 as a whole, if *k* = 1, then once a hypothesis is rejected, it no longer plays a further role and step (iv) above reduces to rejecting any *H_i_*, *i* ∈ *I*, for which *p_i_* ≤ *w_i_*(*I*)*α*. Hence, Algorithm 3 is equivalent to Algorithm 8 (the usual Bonferroni-based graphical approach for FWER control) in that both algorithms will lead to exactly the same rejections when *k* = 1, assuming the same initial weights. When *k* > 1, however, the algorithm becomes more complex and involves maximizing over subsets including *k* − 1 of the previously rejected hypotheses in step (iv). As noted by Romano and Wolf,^18^ intuitively this is because when considering a set of unrejected hypothesis in Algorithm 3, we may have already rejected (hopefully at most) *k* − 1 true null hypotheses. We do not know which of the rejected hypotheses are true, and so we maximize over subsets including at most *k* − 1 of those hypotheses previously rejected. In Appendix B1, we discuss the computational challenges of using Algorithm 3 for large values of *m*, and show how to streamline and operationalize the algorithm. However, in general these modified procedures only give asymptotic control of the *k*-FWER as the sample size of the trial increases.

In Appendix B2, we give some examples of using the generalized graphical approach. We show how it reduces to previous algorithms for *k*-FWER control as special cases, but also how it can have undesirable properties when the testing procedure has a hierarchical structure. The main problem (as demonstrated analytically in Example 4 of Appendix B2) is that if a hypothesis *H_j_* has fewer than *k* donors, its initial significance level will never increase, except for up to *k* − 2 hypotheses via the subprocedure in step (iii). Here, the donors of a hypothesis *H_j_* are the hypotheses that donate (or propagate) their significance levels to *H_j_* if they are rejected. Hence, the generalized graphical approach cannot effectively propagate the significance levels through the graph. We will see further examples of this in the case studies given in Section 6.

**Example 2** (Example of the generalized graphical approach for *k*-FWER control:). We again consider the graph of the diabetes trial given in Figure 1. We control the *k*-FWER for *k* = 2 with *ρ* = .05, with the *P*-values this time given by *P*_1_ = .01, *P*_2_ = .03, *P*_3_ = .02, *P*_4_ = .024. Applying the generalized graphical approach for *k*-FWER control gives the following:

1. Set *I* = {1, 2, 3, 4}.
2. We reject any *H_i_*, *i* ∈ *I*, for which *p_i_* ≤ *w_i_*(*I*)*kα*. Here *w_i_*(*I*) are simply the initial weights and so *w*_1_(*I*) = *w*_2_(*I*) = 0.5 and *w*_3_(*I*) = *w*_4_(*I*) = 0. Since *p*_1_ *< α*, *p*_2_ *< α*, *H*_1_ and *H*_2_ are rejected at this step.
3. We reject *H_i_*, *i* ∈ *I* = {3, 4}, if *p_i_* ≤ min{*w_i_*({1, 3, 4})*, w_i_*({2, 3, 4})}. However, *w*_3_({1,3,4}) = *w*_4_({2,3,4}) = 0 and hence neither *H*_3_ nor *H*_4_ can be rejected.

### 3.3 Existing power comparisons

Romano and Wolf^16^ argue that the augmented procedure is suboptimal compared with their generic stepdown method for *k*-FWER control, since it can only reject at most *k* − 1 hypotheses more compared with a usual FWER-controlling procedure, whereas Algorithm 3 can reject substantially more hypotheses. In their simulation study [16, Section 6], they considered testing the means of a multivariate normal distribution with common correlation *ρ*, where the number of hypotheses *M* = 50 or *M* = 400. They compared a number of different procedures for *k*-FWER control, but the relevant power comparison for our context of graphical approaches is the one between the generalized Holm procedure and the augmented Holm procedure. Their simulation results showed that when *M* = 400, *k* = 10 and *α* ≤ 0.5, the generalized Holm procedure can make a substantially higher number of rejections (up to twice as many) compared with the augmented Holm procedure. However, when *M* = 50 and *k* = 3, the augmented Holm procedure almost always had a higher number of rejections than the generalized Holm procedure.

These findings are corroborated by the simulation results of Dudoit et al.^27^ They also considered testing the means of a multivariate normal distribution, with the number of hypotheses *M* = 24 or *M* = 400. Through simulation, they compared the augmented and generalized Holm and Bonferroni procedures, concluding that the augmented approach tends to be more powerful than the generalized approach “for a broad range of models” [27, Section 6.2.1]. The largest gains in power were when the number of hypotheses was small and a large proportion of the null hypotheses were true. However, for a large number of hypotheses (*M* = 400) and when *α* was relatively large, the generalized approaches was more powerful than the augmented approaches. In many clinical trials, we would be in the setting with a smaller number of hypotheses, and so the augmented approach would be expected to be more powerful. In our case studies in Section 6, we consider power comparisons beyond Bonferrroni or Holm based methods.

## 4 Graphical Approaches for fdp and (Asymptotic) fdr Control

In this section, we consider how to extend the graphical approach for FDP and (asymptotic) FDR control. More formally, the FDP is defined as $\text{FDP}=\frac{V}{\max(R,1)}$, where *R* denotes the total number of rejections. The FDR is then the expectation of the FDP. A multiple testing procedure controls the tail probability of the FDP at level *α* if *P*(FDP *> α*) ≤ *α*, where *α* ∈ [0,1) is a prespecified bound. This is also known as the tail probability for the proportion of false positives^27^ or the false discovery exceedance.^28^ Note that setting *α* = 0 results in control of the FWER at level *α*. In what follows, when we refer to FDP control, we mean controlling this tail probability of the FDP, where we suppress the dependence on *γ* for notational convenience.

### 4.1 Augmented approach for FDP and FDR control

A simple method of controlling the FDP based on a FWER-controlling procedure is given by van der Laan [22, Procedure 2]. This can be applied to give an augmented graphical approach for FDP control, in a similar way to that for *k*-FWER control. A proof that FDP control holds can be found in van der Laan [22, Theorem 2].

**Algorithm 4 (Augmented graphical approach for FDP control).**

(i) *Apply the usual Bonferroni-based graphical FWER procedure given in Algorithm 8. Let R denote the index set of the rejected hypotheses*.
(ii) *Let I denote the index set of any remaining (unrejected) hypotheses. If I is empty, then stop*.
(iii) *Let D be the largest integer satisfying*
  $$\frac{D}{D+|R|}\leq \gamma$$
  *If D* = 0 *then stop; otherwise continue with steps (ii) to (iv) of Algorithm 8 with α replaced by α, until up to D additional (augmented) rejections are made*.

Here *α* ≥ 0 is a constant controlling how many additional rejections are made. As before, *δ* may be greater than *δ*, and can be set very large so that all *D* additional hypotheses are rejected. The choice of *δ* can also be data-dependent, giving an alternative algorithm based on adjusted *P*-values, which does not depend on an explicit choice of *δ*.

**Algorithm 5 (Adjusted augmented graphical approach for FDP control).**

(i) *Calculate the m adjusted P-values $P_{i}^{\text{adj}\;}$ corresponding to the usual Bonferroni-based graphical procedure for FWER control, as detailed in Algorithm 9*.
(ii) *Reject all hypotheses H_i_ with* $P_{i}^{adj}\leq \alpha$.
(iii) *Let I denote the index set of any remaining (unrejected) hypotheses. If I is empty then stop; otherwise order the remaining hypotheses in nondecreasing order*: $P_{\left(1\right)}^{\text{adj}}\leq \ldots \leq P_{\left(II\right)}^{\text{adj}}$
(iv) *Additionally reject up to A* = *min(|I|, D*) *hypotheses H*_(*i*)_ *corresponding to* $P_{\left(1\right)}^{\text{adj}},\ldots ,P_{\left(A\right)}^{\text{adj}}$

If there are ties in the ordering in step (iii), they can be broken by choosing the hypothesis with the smallest index. Algorithm 4 will give the same rejections as Algorithm 5 for *δ* large enough. In addition, the R package gMCP^25^ can straightforwardly be used to implement Algorithm 4 in two stages corresponding to steps (i) and (iii). Hence, we focus on Algorithm 4 in the remainder of the article.

**Example 3** (Example of the augmented graphical approach for FDP control:). We continue the example of the diabetes trial displayed in Figure 1, where this time we aim to control the FDP with *α* = .05 and *δ* = 0.5. Suppose this time the *P*-values are given by *P*_1_ = .01, *P*_2_ = .015, *P*_3_ = .02, *P*_4_ = .024. In step (i), the Bonferroni-based graphical procedure for FWER control would reject *H*_1_ and *H*_2_. We then reject up to *D* additional hypotheses in step (iii), where *D* is the largest integer satisfying *D*/(*D* + 2) ≤ *γ*. Hence if 0 ≤ *γ* < 1/3 we make *D* = 0 additional rejections, if 1/3 ≤ *γ <* 1/2 we make *D* = 1 additional rejection (reject *H*_3_), and if *γ* ≥ 1/2 we make *D* = 2 additional rejections (reject *H*_3_ and *H*_4_).

Although our focus in this article is on controlling the tail probability of the FDP, we note in passing that the augmented procedure for FDP control at level *α* automatically gives asymptotic control of the FDR at level 2*α*. This follows directly from van der Laan [22, Theorem 3]. Hence, applying the augmented graphical approach for FDP control given in Algorithm 4 at prespecified level *α* asymptotically controls the FDR at level 2*α*. Lehmann and Romano^12^ showed that FDP control at level *α* also implies FDR control at level *α* ^*^ = *α*(1 − *γ*) + *α*. Hence, if *α* ^*^ < 2*α*, which implies that *γ < α*/(1 − *γ*), this bound can be used instead, while also yielding finite sample FDR control.

4.2

Generalized graphical approach for asymptotic FDP control

As an alternative method to control the FDP, we can directly apply the generic method for FDP control in Romano and Wolf [18, Algorithm 8.1] to give the following graphical approach.

**Algorithm 6 (Generalized graphical approach for asymptotic FDP control).**

(i) *Let j* = 1 *and k*_1_ = 1
(ii) *Apply the k_j_-FWER procedure given in Algorithm 3, and let R_j_ denote the index set of the hypotheses it rejects*.
(iii) *If* |*R_j_*| *< k_j_*/*γ* − 1*, stop and reject all hypotheses rejected by the k_j_-FWER procedure. Otherwise, let j* = *j* + 1 *and k_j_* = *k_j_*_−1_ + 1*, then return to step (ii)*.

This algorithm was only proven in Romano and Wolf^18^ to give asymptotic FDP control, but they showed empirically that it had good finite control of the FDP. However, since Algorithm 6 is based on the *k*-FWER generalized graphical approach, the same potential problems as described in Appendix B2 will also apply. Finally, we again note in passing that the result of Lehman and Romano^12^ shows that this procedure gives (asymptotic) FDR control at level *α* ^*^ = *α*(1 − *γ*) + *γ*.

**Example 4** (Example of the generalized graphical approach for FDP control:). We continue the example of the diabetes trial displayed in Figure 1, where we aim to control the FDP with *α* = .05 and *δ* = 0.5. Suppose the *P*-values are given by *P*_1_ = .01, *P*_2_ = .015, *P*_3_ = .02, *P*_4_ = .024. In step (ii), applying the FWER procedure results in the rejection of *H*_1_ and *H*_2_. Hence |*R*_1_| = 2 and we stop if *γ <* 1/3. If *γ* ≥ 1/3, then we apply the 2-FWER procedure, which again rejects *H*_1_ and *H*_2_. Hence |*R*_2_| = 2 and we stop if *γ <* 2/3. If *γ* ≥ 2/3, then we apply the 3-FWER procedure, which rejects *H*_1_ and *H*_2_. Since |*R*_3_| = 2 < 3/*γ* − 1 for all *γ <* 1 we would stop at this step.

### 4.3 Existing power comparisons

Romano and Wolf^16^ argue that the augmented procedure for FDP control is suboptimal compared with their generalized method for FDP control, given that both are based on the *k*-FWER controlling procedures. In the simulation results for FDP controlling procedures given in Dudoit et al^27^ and Romano and Wolf,^16^ the augmented and generalized approaches as given above (Algorithms 4 and 6) are not directly compared for Holm (or Bonferroni) based procedures. However, given their simulation results for *k*-FWER control, we might also expect the augmented approach to have a higher power than the generalized approach when the number of hypotheses are small or when the proportion of true null hypotheses is high. We consider such power comparisons in our case studies in Section 6.

## 5 Extensions to The Graphical Approach

The original Bonferroni-based graphical approach of Bretz et al^3^ has been extended in a number of ways.^29^ These extensions can be used in the augmented and generalized procedures for *k*-FWER and FDP control.

### 5.1 Entangled graphs

First, we consider the setting where it is desirable for the graphical procedures to have memory, in the sense that the propagation of significance levels depends on their origin. To achieve this, we can define individual graphs for each relationship and combine them afterward. This is known as an entangled graph, and the algorithm presented in Maurer and Bretz^30^ gives an entangled Bonferroni-based graphical approach.

Hence, we can straightforwardly modify the augmented graphical approaches for *k*-FWER and FDR control for use with entangled graphs. To do so, simply replace Algorithm 8 with the algorithm of Maurer and Bretz.^30^ For the adjusted augmented graphical approaches, replace Algorithm 9 with the algorithm of Maurer and Bretz,^31^ which shows how to calculate adjusted *P*-values for the entangled graph setting. Maurer and Bretz^30^ also showed how to calculate the weights for any intersection hypothesis *H_J_*, *J ⊆ M*, and this weighting strategy satisfies the monotonicity condition given in Equation (2). Hence, we can directly apply this weighting strategy to the generalized graphical approaches for *k*-FWER and FDP control. We give an example of the use of entangled graphs in the case study described in Section 6.2.

### 5.2 Weighted parametric tests

All the procedures so far have been based on weighted Bonferroni tests, which can be conservative. As an alternative, weighted parametric tests can be used if the joint distribution of the *P*-values *p_j_*, *j* ∈ *J*, are known for the intersection hypothesis *H_J_*. In this case, a weighted min-*p* test can be defined.^32,33^ This test rejects *H_J_* if there exists a *j* ∈ *J* such that *p_j_* ≤ *c_J_w_j_*(*J*)*α*, where *c_J_* is the largest constant satisfying

$$P_{H_{j}}\left(\underset{j=1}{\cup }\left\{p_{j}\leq c_{j}w_{j}(J)\alpha \right\}\right)\leq \alpha .$$

If only some of the multivariate distributions of the *P*-values are known, then Bretz et al^24^ and Xi et al^34^ showed how to derive conservative upper bounds on this rejection probability, and hence determine a value for *c_I_*.

The motonocity condition in this setting is

(3) $$c_{J}w_{j}(J)\leq c_{J^{\prime }}w_{j}\left(J^{\prime }\right)\mathrm{and}\text{for}\;\text{all}\;J^{\prime }\subseteq J\subseteq M\text{and}\;j\in J^{\prime }.$$

which implies that rejection thresholds are always more liberal when fewer hypotheses are included in the set. In practice, this condition is often violated when using weighted parametric tests.^24^ If this is the case, then it may be possible to modify the weighting scheme so that Equation (3) holds.^24,34^ If the monotonicity condition does hold, then we can use the weighted parametric tests directly for the augmented and generalized approaches for *k*-FWER and FDP control, with the only change being that *w_i_*(*I*) is replaced by *c_I_w_i_*(*I*). For the adjusted augmented graphical approach, adjusted *P*-values can be constructed for weighted parametric tests.^34^

### 5.3 Group sequential designs

The graphical approach can also be extended to group sequential designs with one or more interim analyses. Under mild monotonicity conditions, Maurer and Bretz^35^ proposed a graphical testing procedure for multiple hypotheses and multiple interim analyses. More formally, consider testing *H*_1_,…,*H_m_* in a group sequential trial at time points *t* = 1,…,*h*. Each *H_i_* has an associated error spending function *a_i_*(*α*,*y*) with information fraction *y* and significance level *κ*. The nominal significance levels are denoted by ${\tilde{\alpha }}_{i,i}(\kappa )$, which are the interim decision boundaries. We assume that these nominal levels satisfy the monotonicity condition ${\tilde{\alpha }}_{i,t}\left(\kappa^{\prime }\right)\geq {\tilde{\alpha }}_{i,t}(\kappa )$ for all *k*′ > *K* (ie, the rejection boundaries are always higher when the total error rate of the design is higher). These conditions hold for many spending functions, including O’Brien-Fleming and Pocock boundaries.^35^ The algorithm presented in Maurer and Bretz^35^ gives a Bonferroni-based graphical test procedure for group sequential designs.

The augmented graphical approaches for *k*-FWER and FDP control can hence be extended to apply to group sequential designs: simply replace Algorithm 8 with the algorithm in Maurer and Bretz.^35^ For the adjusted augmented graphical approach, replace Algorithm 9 with the algorithm of Maurer and Bretz,^31^ which shows how to calculate adjusted *P*-values for the group sequential design setting.

## 6 Case Studies

In this section, we compare and contrast the use of the algorithms for *k*-FWER and FDP control on three clinical case studies covering a broad range of clinical trial applications. In Section 6.1 we revisit an exploratory pharmacodynamic clinical trial to investigate the effect of drug activity at the GABA-A receptor in the brain. In Section 6.2, we revisit a proof-of-concept trial investigating three doses of a new drug against a placebo on multiple biological endpoints related to acute heart failure. Finally, in Section 6.3 we illustrate the proposed approaches for the comparison of three therapies in a confirmatory clinical trial for heart failure patients.

### 6.1 Pharmacodynamic study

**Figure 3:**
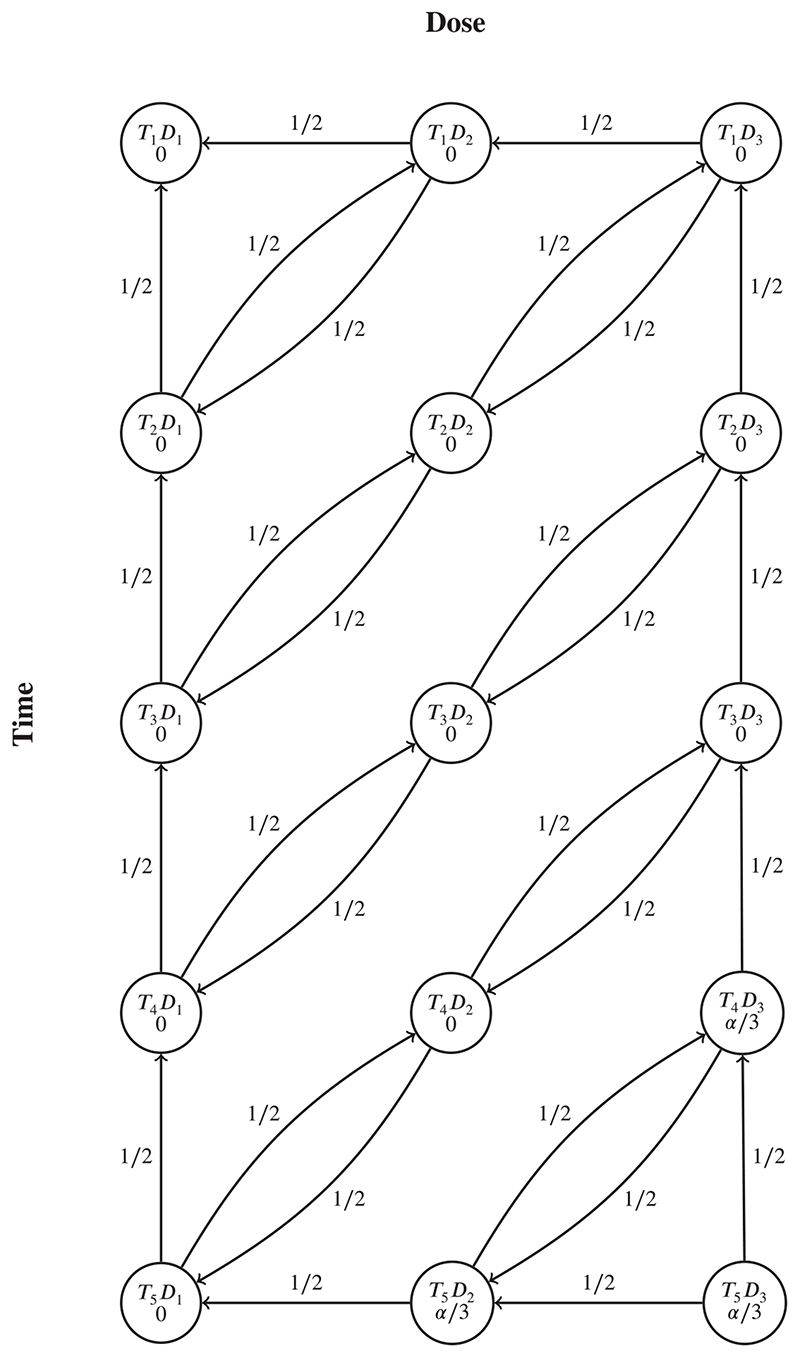
Graph representing the testing strategy for the pharmacodynamic study described in Ferber et al,^36^ with modified initial weights.Here contrast *T_i_D_j_* compares the change from baseline under dose *j* (*j* = 1,2,3) at time point *i* (*i* = 1,…,5) to the corresponding change under placebo

Our first case study is motivated by the exploratory pharmacodynamic clinical study reported by Ferber et al,^36^ which explored the effect of drug activity at the GABA-A receptor in the brain as measured using a quantitative electroencephalogram (qEEG). Three doses of the drug (0.25, 0.5, and 1 mg) were tested as well as a placebo. During the first 15 minutes after the drug was given to each patient, qEEG measurements were taken and afterward subdivided into five time slices of 3 minutes duration. The analysis strategy used a mixed effect linear model to obtain 15 contrasts to formally test. Contrast *T_i_D_j_* compared the change from baseline under dose *j* (*j* = 1,2,3) at time point *i* (*i* = 1,…,5) to the corresponding change under placebo. Figure 3 shows the graph representing the hierarchical testing strategy used for these 15 hypotheses (with modified initial weights, see below), and Table 1 gives the unadjusted *P*-values from the mixed effects linear model for the 15 hypotheses.

Figure 3 shows that the hypotheses *T*_4_ *D*_3_ and *T*_5_ *D*_2_ each only have a single donor hypothesis (*T*_5_ *D*_3_). Hence if they have initial weights of zero (as in the original graph^36^), then they cannot be rejected by the generalized graphical approach for *k*-FWER control with *k* = 2. This then means that no hypotheses can be rejected except for *T*_5_ *D*_3_. Therefore, we first set the initial weights for *T*_4_ *D*_3_,*T*_5_ *D*_2_, and *T*_5_ *D*_3_ to 1/3, with all other weights set equal to zero.

Table 2 shows the resulting rejections for the generalized and augmented graphical *k*-FWER and FDP controlling procedures, with *δ* = 1. Looking first at the *k*-FWER procedures, for *k* = 1 the generalized and augmented graphical procedures both reject the same eight hypotheses, as would be expected. For *k* = 2 and *k* = 3, the augmented procedure rejects 9 and 10 hypotheses, respectively. However, the generalized graphical procedure rejects fewer hypotheses when *k* = 2 and *k* = 3, with only three rejections in both cases. For *k* = 3 this is because all hypotheses have fewer than three donors and hence only those hypotheses with nonzero initial weights can be rejected. This is still the case when *k* = 2, even though all hypotheses (except for *T*_5_ *D*_3_) have two donors, showing that the generalized graphical procedure cannot effectively propagate the significance levels through the graph.

There is a similar pattern for the FDP controlling procedures, which is expected given that they are based on the *k*-FWER controlling procedures. For *γ* = 0.1 the generalized and augmented graphical procedures give the same eight rejections, which are also the same as the *k*-FWER controlling procedures when *k* = 1. For *γ* = 0.2 and *γ* = 0.3, the augmented procedure rejects 10 and 11 hypotheses, respectively. However, again the generalized graphical procedure rejects fewer hypotheses for the larger values of *γ* = 0.2 and *γ* = 0.3, with only three hypotheses rejected. These are the same rejections as the generalized *k*-FWER controlling procedure for *k* > 1, because *k_j_* > 1 in Algorithm 6.

We also consider the setting where all 15 hypotheses have initial weight of 1/15. Table 3 shows the resulting rejections for the generalized and augmented graphical *k*-FWER and FDP controlling procedures, with *δ* = 1. With these new initial weights, the *k*-FWER controlling procedures both reject the same seven hypotheses when *k* = 1 and the same eight hypotheses with *k* = 2. This shows how the generalized graphical procedure can benefit with nonzero initial weights. However, for *k* = 3 the augmented procedure rejects one more hypothesis (*T*_5_ *D*_1_) than the generalized graphical procedure. This is because all hypotheses have fewer than three donors, and hence the weights for the generalized graphical procedure cannot increase—that is, there is no propagation of the significance levels.

Similarly, the FDP controlling procedures both reject the same seven hypotheses when *γ* = 0.1 and the same eight hypotheses when *γ* = 0.2, which are also the same rejections as the *k*-FWER controlling procedures when *k* = 1 and *k* = 2, respectively. However, for *α* = 0.3 the augmented procedure rejects two more hypotheses than the generalized graphical procedure, while the latter only gives the same rejections as when *γ* = 0.2. This is because when *γ* = 0.3, *k_j_* > 2 in Algorithm 6 and there is no propagation of the significance levels.

### 6.2 The Pre-RELAX-AHF trial

Our second case study is a proof-of-concept trial called the Preliminary study of RELAXin in Acute Heart Failure (Pre-RELAX-AHF).^37^ The trial compared three doses of relaxin against a placebo on multiple biological endpoints related to acute heart failure. Given that this was a proof-of-concept trial, less stringent error rates can be used when adjusting for multiplicity.

One criterion for recommending the treatment for further testing is to show an effect on the majority of multiple endpoints. Following Davison et al,^38^ we consider a subset of nine endpoints. We focus on the 30 μg/kg/day dose of relaxin treatment, which showed efficacy on six of these endpoints when compared with placebo, using one-sided (uncorrected) *P*-values with *α* = .1. In what follows, we call the 30 μg/kg/day dose of relaxin treatment the experimental treatment, and the placebo the control treatment.

Since the experimental treatment was declared efficacious in six out of nine endpoints in the pre-RELAX-AHF trial, we consider a trial design where it is required to reject at least six out of nine hypotheses to declare success. Calling these the primary hypotheses, we then add a hierarchical structure to this trial by supposing that we also test secondary hypotheses if at least six out of the nine primary hypotheses were rejected. Hence, we have a family of primary hypotheses 𝓕_1_ = (*H*_1_, … *, H*_9_) corresponding to testing the experimental treatment against the control across the nine endpoints, and a family of secondary hypotheses 𝓕_2_.

We can represent this six out of nine gatekeeping procedure using entangled graphs, which were described in Section 5.1. More precisely, we can define gatekeeping graphs for all $\left(\begin{array}{l} 9\\ 6 \end{array}\right)=84$ possible subsets of six primary hypotheses and then entangle them.^30^ We perform a Holm procedure 𝓗𝓟_*l*_ on six hypotheses for each of the 84 subsets of size 6, which we denote *J_l_*, *l* = 1,…,84. The full significance level *α* is passed on to 𝓕_2_ if all six hypotheses in 𝓕_1*l*_ = {*H_i_* ∶ *i* ∈ *J_l_*} are rejected. The testing procedure is given by the entangled graph ≥ (***c***, 𝓗𝓟_*l*_; *l* = 1, … , 84) where *c_i_* = 1/84 for *i* = 1,…,84.

This is equivalent to the following testing strategy: the usual Holm procedure is performed on the nine hypotheses in 𝓕_1_ at level *α* until any six of these hypotheses are rejected. The remaining primary and secondary hypotheses are then tested using the weights given in Table 4, which depend on the number |*I*| of unrejected hypotheses in 𝓕_1_. For simplicity, in what follows we suppose that 𝓕_2_ consists of a single hypothesis *H*_10_ (which could, eg, represent a composite safety endpoint). We can then use the weights given in Table 4 in the *k*-FWER and FDP controlling graphical procedures.

In our simulation study, for the primary hypotheses 𝓕_1_ we follow Delorme et al^39^ and take the empirical means and standard errors of the endpoints as the true parameter values for the experimental (E) and control (C) treatments. The numerical values of the means *α^C^*, *α^E^* and standard deviations *μ_C_*, *μ_E_* are given in Appendix C. We assume that the distributions of the observed means of the endpoints for the experimental and control treatments follow a multivariate normal distribution: ${\bar{X}}^{G}∼N\left(\mu^{G},{\text{Σ}}_{G}\right)$ where *G* ∈ {*C*,*E*} and Σ_*G*_ = diag(*σ_G_*)Σ(*ρ*)diag(*σ_G_*). Here diag(*σ_G_*) is a diagonal matrix with the *i*th diagonal element equal to $\mu_{i}^{G}$, and Σ(*ρ*) is a correlation matrix with ones on the diagonal and *ρ* on all off-diagonal terms. The test statistic for endpoint *i* is given by $T_{i}=\hat{Var}{\left({\bar{X}}_{i}^{E}-{\bar{X}}_{i}^{c}\right)}^{-1/2}{\bar{X}}_{i}^{E}-{\bar{X}}_{i}^{c}$, which is compared with a *t*-distribution. The estimator of the variance of the difference between the means, as well as the appropriate degrees of freedom for the *t*-distribution are given by Delorme et al^39^ and implemented in their R package rPowerSampleSize.^40^ For the secondary hypothesis *H*_10_, for simplicity we assume that the test statistic *T*_10_ follows a normal distribution with mean 3 and variance 1, and is independent of the test statistics for 𝓕_1_.

Table 5 gives the marginal power to reject each hypothesis *H*_1_,… *H*_10_, calculated using 10^4^ trial replications, with *α* = .1 and *δ* = 1. The results show that in all scenarios, the augmented procedure has an equal or higher power to reject each of the hypotheses *H*_1_,…,*H*_10_. For the primary hypotheses *H*_1_,…,*H*_5_ and *H*_8_, this is especially noticeable for the *k*-FWER controlling procedures when *k* = 2 and *k* = 3. For hypothesis *H*_9_, the augmented procedures have a substantially higher power compared with the generalized graphical procedure (except for when controlling the usual FWER). However, *H*_9_ is actually a true null hypothesis (with $\mu_{9}^{C}=\mu_{9}^{E}=0.07)$) and so this implies a higher type I error rate for *H*_9_ when using the augmented procedure. In fact the type I error rate for *H*_9_ is below or equal to the nominal 10% in all scenarios for the generalized graphical procedures. Finally, for the secondary hypothesis *H*_10_ (which has an initial weight of zero), we see that the power decreases as *k* and *α* increases for the *k*-FWER and FDP controlling generalized graphical procedures, respectively (in particular, the power is only 6% when *γ* = 0.3 for the latter procedure). Again this shows that in contrast to the augmented procedures, the generalized graphical approaches do not effectively propagate the significance levels when there is a hierarchical structure in the hypotheses.

### 6.3 Atmosphere study

Our final case study is motivated by the confirmatory ATMOSPHERE study^41^ in patients with heart failure. As described in Maurer and Bretz,^31^ the trial compared three therapies: aliskiren monotherapy (A), enalapril monotherapy (E), and aliskiren/enalapril combination therapy (C). This resulted in three single primary hypotheses (*H*_1_,*H*_2_,*H*_3_) and two families of secondary hypotheses (𝓗_4_, 𝓗_5_):

*H*_1_: nonsuperiority of C vs E

*H*_2_: inferiority of A vs E

*H*_3_: nonsuperiority of A vs E

𝓗_4_ = {*H*_41_ *, H*_42_}: two secondary endpoints for comparing C vs E

𝓗_5_ = {*H*_51_ *, H*_52_}: two secondary endpoints for comparing A vs E

**Figure 4:**
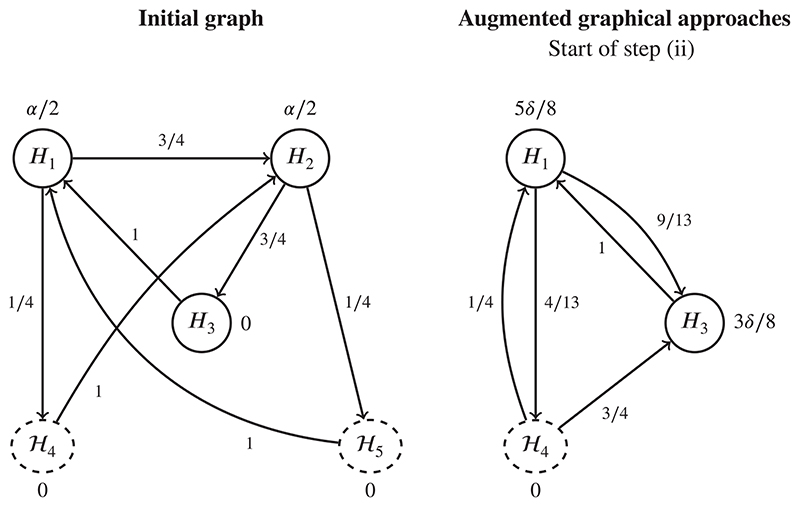
The graph on the left-hand side was used for the ATMOSPHERE study, as presented in Maurer and Bretz.^31^ The graph on the right-hand side is the updated graph at the start of step (ii) in the augmented graphical approach for either *k*-FWER or FDP control, after *H*_2_,*H*_51_, and *H*_52_ have been rejected. FDP, false discovery proportion; FWER, familywise error rate

The graph on the left-hand side in Figure 4 shows the graphical test procedure used in Maurer and Bretz^31^ to analyse the trial. Note that if all individual null hypotheses in 𝓗_4_ or 𝓗_5_ are rejected, the local significance level is propagated to the remaining hypotheses. For simplicity, we apply a Holm procedure within each of the two secondary families 𝓗_4_ and 𝓗_5_. Following Reference 31, suppose we observe the (hypothetical) unadjusted *P*-values *P*_1_ = .1, *P*_2_ = .007, *P*_3_ = .05, *P*_41_ = .0015, *P*_42_ = .04, *P*_51_ = .0031, and *P*_52_ = .001.

Consider first controlling the *k*-FWER with *k* = 2 and *α* = .025. For the augmented graphical approach (given in Algorithm 1), in step (i) the Bonferroni-based graphical procedure for FWER control would reject *H*_2_, *H*_51_, and *H*_52_. The updated graph used at the start of step (ii) is shown in the right-hand side of Figure 4, where *α* has been replaced by *δ*. Supposing that *δ* = 0.5, step (ii) of the algorithm rejects *H*_3_. Since we have made one additional (augmented) rejection, at this point we stop. As for the generalized graphical approach for *k*-FWER control (given in Algorithm 3), in step (ii) we would only reject *H*_2_. Since the number of rejections |*R*| = *k* − 1, we stop at this point.

Now consider controlling the FDP with *γ* = 0.3. For the augmented graphical approach (given in Algorithm 4), in step (i) we reject *H*_2_, *H*_51_, and *H*_52_ like before. In step (ii), we can reject one additional hypothesis, and hence we reject *H*_3_ and then stop. Finally, for the generalized graphical approach (given in Algorithm 6), we first apply the usual Bonferroni-based graphical procedure for FWER control, which rejects *H*_2_, *H*_51_, and *H*_52_. Since |*R*_1_| > 1/*γ* − 1, we then apply the 2-FWER procedure which (as above) only rejects *H*_2_. Since |*R*_2_| < 2/*γ* − 1, we stop and only reject *H*_2_.

## 7 Discussion

In this article, we have showed how to generalize the graphical approach of hypothesis testing^3^ so that the *k*-FWER or the FDP can be controlled. By applying the methodology of Romano and Wolf^18^ and van der Laan,^22^ we have proposed generalized and augmented graphical approaches for both *k*-FWER and FDP control (as well as an augmented procedure for asymptotic FDR control). Crucially, these approaches respect the hierarchical structure of the underlying multiple testing procedure given by the graphical weighting strategy. We have also applied the proposed graphical approaches to three real-life case studies covering a broad range of clinical trial applications.

Our recommendation is that the augmented graphical approaches should be used instead of the generalized graphical approaches. First, the generalized graphical approach for *k*-FWER control has the undesirable property that if a hypothesis *H_j_* has fewer than *k* donors, its initial significance level will not increase. Hence, the generalized graphical approach cannot effectively propagate the significance levels through the graph. The case studies in Section 6 show how this can have a detrimental effect on the power of the generalized graphical approach—the power to reject hypotheses with fewer than *k* donors can actually decrease as *k* increases. Since the generalized graphical approach for FDP control is based on the generalized graphical approach for *k*-FWER control, a similar problem occurs.

By contrast, the augmented graphical approach is able to propagate significance levels to all hypotheses that have fewer than *k* donors. As a consequence, the power of the augmented graphical approach for *k*-FWER control and FDP control increases as *k* and *γ* increase (respectively). Importantly, in all of the case studies in Section 6, the augmented graphical approach had a higher power (or rejected at least as many hypotheses) compared with the generalized graphical approach. These results are backed up by existing power comparisons for the generalized and augmented Holm procedure^16,27^ when testing a relatively small number of hypotheses.

The research for this article was motivated by clinical trial applications ranging from early to late drug development, as illustrated by the case studies in Section 6. Outside of the context of clinical trials and the graphical weighting strategy of Bretz et al,^3^ another area of application is testing hypotheses in a directed acyclic graph (DAG) for use in gene set analysis, as proposed by Meijer and Goeman.^42^ The authors presented a top-down method that strongly controls the FWER, and by considering the genes and gene sets as nodes in a DAG, the method allows testing for simultaneous testing of both significant gene sets and individual genes. The testing procedure starts with an initial weight for each of the leaf nodes (ie, nodes without any descendants), and an iterative weighting procedure is used to update the weights for all the other nodes in the graph. These weights also satisfy the monotonicity condition given in Equation (2), and so suitably modified versions of the augmented and generalized graphical approaches could be used in this setting.

As future work, it would be desirable to derive adjusted *P*-values for all of the proposed procedures, especially for the augmented graphical approaches. This would involve extending the results of van der Laan,^22^ who showed how to calculate adjusted *P*-values for their augmented approach. Finally, the initial motivation for this article came from considering the generalized closure principle,^43^ which was applied to derive stepup procedures for *k*-FWER control. The usual graphical approach for FWER corresponds to defining a shortcut closed testing procedure.^3^ It would be interesting to formalize a similar link between the generalized graphical approach for *k*-FWER control and the generalized closure principle.

## Supplementary Material

Supplementary Material

## Figures and Tables

**Table 1:** Table of *P*-values for the pharmacodynamic study of Ferber et al^36^

Dose	Time				
	*T*_1_	*T*_2_	*T*_3_	*T*_4_	*T*_5_
*D*1	0.7808	0.0600	0.0137	0.0724	0.0162
*D2*	0.9433	0.0053	6.5 × 10 ^−6^	2.8 × 10 ^− 6^	9.1 × 10 ^−8^
*D*3	0.9993	1.0 × 10^−5^	1.7×10^−11^	4.2×10^−12^	8.1×10^−13^

**Table 2:** Rejected hypotheses for the pharmacodynamic study of Ferber et al,^36^ with initial weights of 1/3 for *T*_4_ *D*_3_,*T*_5_ *D*_2_,and *T*_5_ *D*_3_

Procedure		Rejected hypotheses
*k*-FWER		
Generalized	*k* = 1	*T*_2_ *D*_3_,*T*_3_ *D*_2_,*T*_3_ *D*_3_,*T*_4_ *D*_2_,*T*_4_ *D*_3_,*T*_5_ *D*_1_,*T*_5_ *D*_2_,*T*_5_ *D*_3_
	*k* = 2	*T*_4_ *D*_3_,*T*_5_ *D*_2_,*T*_5_ *D*_3_
	*k* = 3	*T*_4_ *D*_3_,*T*_5_ *D*_2_,*T*_5_ *D*_3_
Augmented	*k* = 1	*T*_2_ *D*_3_,*T*_3_ *D*_2_,*T*_3_ *D*_3_,*T*_4_ *D*_2_,*T*_4_ *D*_3_,*T*_5_ *D*_1_,*T*_5_ *D*_2_,*T*_5_ *D*3
	*k* = 2	*T*_2_ *T*_3_,*T*_3_ *T*_2_,*T*_3_ *T*_3_,*T*_4_ *T*_1_,*T*_4_ *T*_2_,*T*_4_ *T*_3_,*T*_5_ *T*_1_,*T*_5_ *T*_2_,*T*_5_ *T*_3_
	*k* = 3	*T*_2_ *T*_3_,*T*_3_ *T*_1_,*T*_3_ *T*_2_,*T*_3_ *T*_3_,*T*_4_ *T*_1_,*T*_4_ *T*_2_,*T*_4_ *T*_3_,*T*_5_ *T*_1_,*T*_5_ *T*_2_,*T*_5_ *T*_3_
FDP		
Generalized	*γ* = 0.1	*T*_2_ *T*_3_,*T*_3_ *T*_2_,*T*_3_ *T*_3_,*T*_4_ *T*_2_,*T*_4_ *T*_3_,*T*_5_ *T*_1_,*T*_5_ *T*_2_,*T*_5_ *T*_3_
	*γ* = 0.2	*T*_4_ *T*_3_,*T*_5_ *T*_2_,*T*_5_ *T*_3_
	*γ* = 0.3	*T*_4_ *T*_3_,*T*_5_ *T*_2_,*T*_5_ *T*_3_
Augmented	*γ* = 0.1	*T*_2_ *T*_3_,*T*_3_ *T*_2_,*T*_3_ *T*_3_,*T*_4_ *T*_2_,*T*_4_ *D*_3_,*T*_5_ *D*_1_,*T*_5_ *D*_2_,*T*_5_ *D*_3_
	*γ* = 0.2	*T*_2_ *T*_3_,*T*_3_ *T*_1_,*T*_3_ *T*_2_,*T*_3_ *T*_3_,*T*_4_ *T*_1_,*T*_4_ *T*_2_,*T*_4_ *T*_3_,*T*_5_ *T*_1_,*T*_5_ *T*_2_,*T*_5_ *T*_3_
	*γ* = 0.3	*T*_2_ *T*_2_,*T*_2_ *T*_3_,*T*_3_ *T*_1_,*T*_3_ *T*_2_,*T*_3_ *T*_3_,*T*_4_ *T*_1_,*T*_4_ *T*_2_,*T*_4_ *T*_3_,*T*_5_ *T*_1_,*T*_5_ *T*_2_,*T*_5_ *T*_3_

Abbreviations: FDP, false discovery proportion; FWER, familywise error rate.

**Table 3:** Rejected hypotheses for the pharmacodynamic study of Ferber et al36 with initial weights of 1/15 for each hypothesis

Procedure		Rejected hypotheses
*k*-FWER		
Generalized	*k* = 1	*T*_2_ *D*_3_,*T*_3_ *D*_2_,*T*_3_ *D*_3_,*T*_4_ *D*_2_,*T*_4_ *D*_3_,*T*_5_ *D*_2_,*T*_5_ *D*_3_
	*k*=2	*T*_2_ *D*_2_,*T*_2_ *D*_3_,*T*_3_ *D*_2_,*T*_3_ *D*_3_,*T*_4_ *D*_2_,*T*_4_ *D*_3_,*T*_5_ *D*_2_,*T*_5_ *D*_3_
	*k =* 3	*T*_2_ *D*_2_,*T*_2_ *D*_3_,*T*_3_ *D*_2_,*T*_3_ *D*_3_,*T*_4_ *D*_2_,*T*_4_ *D*_3_,*T*_5_ *D*_2_,*T*_5_ *D*_3_
Augmented	*k*= 1	*T*_2_ *D*_3_,*T*_3_ *D*_2_,*T*_3_ *D*_3_,*T*_4_ *D*_2_,*T*_4_ *D*_3_,*T*_5_ *D*_2_,*T*_5_ *D*_3_
	*k =* 2	*T*_2_ *D*_3_,*T*_3_ *D*_2_,*T*_3_ *d*_3_,*T*_4_ *D*_2_,*T*_4_ *D*_3_,*T*_5_ *D*_1_,*T*_5_ *D*_2_,*T*_5_ *D*_3_
	*k*=3	*T*_2_ *D*_2_,*T*_2_ *D*_3_,*T*_3_ *D*_2_,*T*_3_ *D*_3_,*T*_4_ *D*_2_,*T*_4_ *D*_3_,*T*_5_ *D*_1_,*T*_5_ *D*_2_,*T*_5_ *D*_3_
FDP		
Generalized	*γ =* 0.1	*T*_2_, *D*_3_, *T*_3_, *D*_2_, *T*_3_, *D*_3_, *T*_4_,*D*_2_, *T*_4_ *D*_3_, *T*_5_, *D*_2_, *T*_5_,*D*_3_
	*γ =* 0.2	*T*_2_ *D*_2_,*T*_2_ *D*_3_,*T*_3_ *d*_2_,*T*_3_ *D*_3_,*T*_4_ *D*_2_,*T*_4_ *D*_3_,*T*_5_ *D*_2_,*T*_5_ *D*_3_
	*γ =* 0.3	*T* 2 *D*_2_, *T*_2_, *D*_3_, *T*_3_, *D*_2_, *T*_3_, *D*_3_, *T*_4_, *D*_2_, *T*_4_, *D*_3_, *T*_5_, *D*_2_, *T*_5_, *D*_3_
Augmented	*γ =* 0.1	*T*_2_ *D*_3_,*T*_3_ *D*_2_,*T*_3_ *d*_3_,*T*_4_ *D*_2_,*T*_4_ *D*_3_,*T*_5_ *D*_2_,*T*_5_ *D*_3_
	*γ =* 0.2	*T*_2_ *D*_3_,*T*_3_ *D*_2_,*T*_3_ *D*_3_,*T*_4_ *D*_2_,*T*_4_ *D*_3_,*T*_5_ *D*_1_,*T*_5_ *D*_2_,*T*_5_ *D*_3_
	*γ =* 0.3	*T*_2_ *D*_2_,*T*_2_ *D*_3_,*T*_3_ *d*_1_,*T*_3_ *D*_2_,*T*_3_ *D*_3_,*T*_4_ *D*_2_,*T*_4_ *D*_3_,*T*_5_ *D*_1_,*T*_5_ *D*_2_,*T*_5_ *D*_3_

Abbreviations: FDP, false discovery proportion; FWER, familywise error rate.

**Table 4:** Table ofweights for the entangled graph procedure used to analyse the trial based on Pre-RELAX-AHF

|*I*|	Weight for each hypothesis in 𝓕_1_	Weight for 𝓕_2_ = {*H*_l0_}
* >*3	1/|*I*|	0
3	83/252	1/84
2	11/24	1/12
1	2/3	1/3
0	–	1


*Note:* Here I*I*| denotes the number of unrejected hypotheses in F_1_.

**Table 5:** Simulated marginal powers to reject hypotheses *H1,... H*w,with *a =* .1 and the distribution of the test statistics for T*i = *(H1,..., H9* *)* based on the Pre-RELAX-AHF trial reported by Teerlink et al37

Procedure		Marginal power									
		*H*1	*H*2	*H*3	*H*4	*H*5	*H*6	*H*7	*H*8	*H*9	*H*10
*k*-FWER											
*k*=1	Generalized	95	89	72	78	85	100	100	62	6	64
	Augmented	95	89	72	78	85	100	100	62	6	64
*k*=2	Generalized	97	93	79	84	90	100	100	70	9	60
	Augmented	98	97	90	92	95	100	100	87	65	84
*k*=3	Generalized	98	95	81	86	92	100	100	73	10	42
	Augmented	100	99	96	97	98	100	100	95	87	95
FDP											
*γ =* 0.1	Generalized	95	89	72	78	85	100	100	62	8	61
	Augmented	95	89	72	78	85	100	100	63	38	65
*γ =* 0.2	Generalized	95	92	78	83	89	100	100	70	9	50
	Augmented	96	92	82	86	90	100	100	77	52	73
*γ =* 0.3	Generalized	96	93	80	85	90	100	100	72	10	6
	Augmented	97	94	86	88	92	100	100	83	58	83

*Note*: Results are based on 104 independent trial replications.

Abbreviations: FDP, false discovery proportion; FWER, familywise error rate.

## Data Availability

All of the data that support the findings of this study are available within the article itself. Code to reproduce the results of Section 6 can be found at https://github.com/dsrobertson/graphical-approach.
