# Supplementary material for: Graphical approaches for the control of generalized error rates
Source: Stat Med. Author manuscript; Available in PMC 2021 Dec 16. (PMC7612110; doi:10.1002/sim.8595)
Supplement: Supplementary Material [file EMS140489-supplement-Supplementary_Material.pdf]

APPENDIX A. GRAPHICAL WEIGHTING STRATEGY AND WEIGHTED BONFERRONI TEST FOR FWER CONTROL

For a given index set $J \subseteq M$, let $J^c = M \backslash J$ denote the set of indices not contained in J

Algorithm 7 (*Graphical weighting strategy [24], Algorithm 1*).

(i) *Set* $I = M$

(ii) *Select* $j \in J^c$ *and remove* H_j

(iii) *Update the graph:*

$$I \to I \setminus \{j\}, J^c \to J^c \setminus \{j\}$$

$$w_l(I) \to \begin{cases} w_l(I) + w_j(I) g_{jl} & l \in I \\ 0 & \text{otherwise} \end{cases}$$

$$g_{lh} \to \begin{cases} \frac{g_{lh} + g_{lj} g_{jh}}{1 - g_{lj} g_{jl}} & l, h \in I,\ l \neq h,\ g_{lj} g_{jl} < 1 \\ 0 & \text{otherwise} \end{cases}$$

(iv) *If* $|J^c| \geq 1$, *go to step (ii); otherwise set* $w_l(J) = w_l(I), l \in J$ *and stop.*

The weights $w_j(J), j \in J$, generated by this procedure are unique,[3] and in particular do not depend on which order the hypotheses $H_j, j \in J^c$ are removed in Algorithm 7.

Algorithm 8 (*Bonferroni-based graphical test for FWER control [24], Algorithm 2*).

(i) *Set* $I = M$

(ii) *Select a* $j \in I$ *such that* $p_j \leq w_j(I)\alpha$ *and reject* H_j; *otherwise stop.*

(iii) *Update the graph:*

$$I \to I \setminus \{j\}$$
$$w_l(I) \to \begin{cases} w_l(I) + w_j(I)g_{jl} & l \in I \\ 0 & \text{otherwise} \end{cases}$$
$$g_{lh} \to \begin{cases} \frac{g_{lh}+g_{lj}g_{jh}}{1-g_{lj}g_{jl}} & l, h \in I,\ l \neq h,\ g_{lj}g_{jl} < 1 \\ 0 & \text{otherwise} \end{cases}$$

(iv) *If* $|I| \geq 1$*, go to step (ii); otherwise stop.*

The final decisions of the algorithm do not depend on which order the hypotheses are rejected. For example, step (ii) above could be replaced by choosing $j = \arg\min_{i \in I}\{p_i/w_i(I)\}$.

Algorithm 9 (*Adjusted P-values [3], Algorithm 2*).

(i) *Set* $I = M$ *and* $p_{\max} = 0$
(ii) *Let* $j = \arg\min_{i \in I} p_i/w_i(I)$
(iii) *Calculate* $p_j^{\text{adj}} = \max\{p_j/w_j(I), p_{\max}\}$ *and set* $p_{\max} = p_j^{\text{adj}}$.
(iv) *Update the graph:*

$$I \to I \setminus \{j\}$$
$$w_l(I) \to \begin{cases} w_l(I) + w_j(I)g_{jl} & l \in I \\ 0 & \text{otherwise} \end{cases}$$
$$g_{lh} \to \begin{cases} \frac{g_{lh}+g_{lj}g_{jh}}{1-g_{lj}g_{jl}} & l, h \in I,\ l \neq h,\ g_{lj}g_{jl} < 1 \\ 0 & \text{otherwise} \end{cases}$$

(v) *If* $|I| \geq 1$*, go to step (ii); otherwise stop.*
(vi) *Reject all hypotheses* H_j *with* $p_j^{\text{adj}} \leq \alpha$

APPENDIX B. FURTHER RESULTS ON THE GENERALIZED GRAPHICAL APPROACH

B.1 Streamlined and operative versions of the generalized graphical approach

Apart from special cases, applying the generalized graphical approach given in Algorithm 3 can be computationally intensive for larger values of m, particularly since the weights $w_i(K)$ need to be calculated using Algorithm 7. For large values of m, we can directly apply the streamlined version of the general stepdown method for controlling the k-FWER in Romano and Wolf [18, Algorithm 4.2] to give a streamlined version of Algorithm 3. The way this version works is to avoid minimizing over all subsets of size $k-1$ of previously rejected hypothesis, and only consider the least significant $k-1$ of the previous rejections. Note that this only gives asymptotic control of the k-FWER (as the sample size of the trial increases).

Algorithm 10 (Streamlined graphical approach for k-FWER control). *Given an index set R of rejected hypotheses, let* $p_{1:R} \leq p_{2:R} \leq \dots \leq p_{|R|:R}$ *denote the ordered P-values, with corresponding hypotheses* $H_{1:R}, H_{2:R}, \dots, H_{|R|:R}$*. Denote by* $\{r_1, \dots, r_{|R|}\}$ *the permutation of* $\{1, \dots, |R|\}$ *that gives this ordering, so that* $p_{1:R} = p_{r_1}, \dots, p_{|R|:R} = p_{r_{|R|}}$*. The streamlined algorithm is the same as Algorithm 3, except that at step (iv) we now reject any* H_i*,* $i \in I$ *for which* $p_i \leq w_i(K)k\alpha$*, where* $K = I \cup \{r_{|R|-k+2}, \dots, r_{|R|}\}$*.*

The streamlined version only gives asymptotic control of the k-FWER, but involves no minimization over any subsets. In order to get closer to the original, exact algorithm while still retaining computational feasibility, as a compromise we can use the operative method proposed in Romano and Wolf [18, Remark 3.3]. Consider that to compute the critical value

in step (iv) of Algorithm 3, one has to evaluate $\binom{|R|}{k-1}$ weights in order to choose the minimum. The operative method maximizes over subsets not necessarily of the entire index set R of previously rejected hypotheses, but only for some number B least significant hypotheses so far. More precisely, we have the following algorithm:

Algorithm 11 (Operative graphical approach for k-FWER control). *Pick a user-specified number $N_{\max}$ and let B be the largest integer for which $\binom{B}{k-1} \leq N_{\max}$. The operative method is the same as Algorithm 3, except that step (iv) rejects any H_i, $i \in I$ for which*

$$p_i \leq \min_{J \subseteq \{r_{\max\{1,|R|-B+1\}},\ldots,r_{|R|}\},|J|=k-1} \{w_i(K) : K = I \cup J\} k\alpha$$

When $B \geq |R|$ we maximize over all subsets of R of size $k-1$ like in the original algorithm, while the streamlined algorithm is a special case of the operative method where $N_{\max} = 1$ and hence $B = k-1$.

B.2 Examples of the generalized graphical approach

Example 5 (Generalized Weighted Bonferroni:). Suppose each vertex on the graph is unconnected, that is, $g_{ij} = 0$ for all $i,j \in M$. Algorithm 7 implies that $w_j(J) = w_j(M)$, $j \in J$ for all $J \subseteq M$. Hence the inequality in step (iv) of Algorithm 3 is simply $p_i \leq w_i(M)k\alpha$ and so there is no further testing after step (ii), unless $\delta > 0$ and $|R| < k-1$. Thus when $\delta = 0$, Algorithm 3 is exactly the same as the generalized weighted Bonferroni procedure in Section 2 with $w_i = w_i(M)$.

Example 6 (Generalized Holm:). To represent the Holm procedure with m hypotheses, we set the initial weights $w_i(M) = 1/m$ and $g_{ij} = 1/(m-1)$ for all $i, j \in M$, $i \neq j$. Hence using Algorithm 7, we have $w_i(I) = 1/|I|$ for all $i \in I$ and $I \subseteq M$, and the inequality in step (iv) of Algorithm 3 is simply

$$p_i \leq \frac{k\alpha}{|I| + k - 1} = \frac{k\alpha}{m + k - |R| - 1}$$

For $\delta = 0$, this gives identical rejections to the generalized Holm procedure as given in Lehmann and Romano.[12]

Example 7 (Hierarchical testing: fixed sequence test and fallback procedure). In a fixed sequence test, the hypotheses are tested in a prespecified order. This allows each hypothesis to be tested at the full level α while controlling the FWER, with the proviso that if any hypothesis is not rejected then no further testing is allowed. Suppose the prespecified ordering for testing m hypotheses is $H_1 \rightarrow H_2 \rightarrow \ldots \rightarrow H_m$. Hence, we have $g_{ij} = 1$ for $i = 1,\ldots,m-1$ if $j = i+1$ and $g_{ij} = 0$ otherwise.

If we follow the usual fixed sequence test and set $w_1(M) = 1$ and $w_i(M) = 0$, $i = 1,\ldots,m-1$, then only H_1 can be rejected in step (ii) of the Algorithm 3 and hence the algorithm will never proceed to step (iv) since $|R| < k$. Hence, a more natural generalization of the fixed sequence test is to set the initial weights as $w_i(M) = 1/k$ for $i = 1,\ldots,k$ and $w_i(M) = 0$ otherwise. This means that the first k hypotheses will be tested at full level α. However, assume that the first k hypotheses are all rejected (otherwise we proceed to the subprocedure of step (iii) and can only reject up to the first $k-1$ hypotheses). Since $w_i(\{1,\ldots,k-1,k+1,\ldots,i,\ldots,m\}) = 0$ for $i = k+1,\ldots,m$, step (iv) of the algorithm implies that no further hypotheses can then be rejected. For $k > 1$, this generalization of the fixed sequence test has the undesirable property that only the first k hypotheses can ever be tested, even when using the subprocedure of step (iii) with $\delta > 0$.

A similar issue occurs when generalizing the fallback procedure,[44] which is a modification of the fixed sequence procedure where the initial weights $w_i(M) > 0$ for all $i \in M$. Applying Algorithm 3, suppose (without loss of generality, by relabeling the hypothesis labels) that the hypotheses $H_1,\ldots,H_k$ are all rejected at step (ii). However, since $w_i(I \cup \{1,\ldots,k-1\}) = w_i(M)$ for all $i \in I$ and $I \subseteq \{k+1,\ldots,m\}$, step (iv) implies that the hypotheses H_i, $i = k+1,\ldots,m$, are also tested at significance level $w_i(M)k\alpha$. So for $k > 1$, this generalization of the fallback procedure has the undesirable property that rejecting hypotheses does not lead to an increase in the significance levels of the remaining hypotheses, except via the subprocedure of step (iii) when $\delta > 0$ (but even then, the propagation is limited to at most $k-1$ hypotheses).

Example 8 (Hypotheses with fewer than k donors:). We can generalize the previous example to any graph where any hypothesis has fewer than k donors, where the donors of a hypothesis H_j are the hypotheses that donate (or propagate) their significance levels to H_j if they are rejected. More formally, we denote the donors of hypothesis H_j by $\text{do}(H_j) = \{H_i : g_{ij} > 0\}$. Note that two hypotheses H_i and H_j can be donors to each other.

If a hypothesis H_j in a graph has fewer than k donors, then applying the generalized graphical approach has the undesirable property that the initial significance level for H_j can never increase, even if all its donors are rejected (except

for up to $k-2$ hypotheses via the subprocedure in step (iii)). To see this, suppose $\delta = 0$ and all donors of H_j have been rejected (and $|R| \geq k$, or else there is no propagation). In step (iv) of Algorithm 3, since $|\text{do}(H_j)| \leq k-1$ then

$$\min_{J \subseteq R, |J|=k-1} \{w_i(K) : K = I \cup J\} k\alpha \leq w_i \left(I \cup \text{do}(H_j)\right) k\alpha = w_i(M) k\alpha.$$

Hence H_j is tested using the initial weights in step (iv). In particular, this means that if a hypothesis with fewer than k donors has an initial weight of zero, then it can never be rejected (except possibly via the subprocedure in step (iii)). This can be an undesirable property to have in a testing procedure which has a hierarchical structure, as we will see further in the case studies in Section 6.

As an example, consider the graph for the diabetes trial shown in Figure 1, and suppose we wish to control the k-FWER for $k = 2$. Since the secondary hypotheses H_3 and H_4 only have one donor each (hypotheses H_1 and H_2, respectively) and start with a weight of zero, they will never be rejected even if more than one of the primary hypotheses H_1 and H_2 are rejected.

APPENDIX C. PARAMETER VALUES FOR THE PRE-RELAX-AHF TRIAL

In our simulation study, for the primary hypotheses $H_1, \ldots, H_9$ we take the empirical means and standard errors of the endpoints as the true parameter values for the experimental (E) and control (C) treatments. The numerical values of the means μ^C, μ^E and standard deviations σ_C, σ_E are given in Delorme et al[39] and reproduced below:

$$\begin{aligned}
\mu^C &= (0.23, 1679, 0.79, -12, 44.2, 0.828, 0.857, 0.13, 0.07)\\
\mu^E &= (0.4, 2567, 0.88, -10.2, 47.9, 0.974, 1, 0.21, 0.07)\\
\sigma_C &= (\sqrt{0.23(1-0.23)}, 2556, \sqrt{0.79(1-0.79)}, 7.3, 14.2, \sqrt{0.828(1-0.828)}, \sqrt{0.857(1-0.857)},\\
&\quad \sqrt{0.13(1-0.13)}, \sqrt{0.07(1-0.07)})\\
&= (0.421, 2556, 0.407, 7.3, 14.2, 0.377, 0.350, 0.336, 0.255)\\
\sigma_E &= (\sqrt{0.4(1-0.4)}, 2898, \sqrt{0.88(1-0.88)}, 6.1, 10.1, \sqrt{0.974(1-0.974)}, 10^{-12}, \sqrt{0.21(1-0.21)},\\
&\quad \sqrt{0.07(1-0.07)})\\
&= (0.490, 2898, 0.325, 6.1, 10.1, 0.159, 10^{-12}, 0.407, 0.255)
\end{aligned}$$
